# Estrogen Receptor Gene (ESR1) PVUII and XBAI Polymorphisms and Bone Mineral Density in Kazakh Women

**DOI:** 10.5195/cajgh.2013.100

**Published:** 2014-01-24

**Authors:** Ainur Akilzhanova, Zhannur Abilova, Akbota Aitkulova, Zaida Zhumatova, Gulbanu Akilzhanova, Elena Zholdybayeva, Kuvat Momynaliev

**Affiliations:** 1 Center for Life Sciences, Nazarbayev University, Astana, Kazakhstan; 2 National Center for Biotechnology, Almaty, Kazakhstan; 3 City Hospital #1, Pavlodar, Kazakhstan; 4 Regional Perinatal Center, Pavlodar, Kazakhstan

**Keywords:** osteoporosis, bone mineral density, genetic risk factors, Kazakhstan

## Abstract

**Introduction:**

Osteoporosis is a common age-related disease that is strongly influenced by genetics. Polymorphisms of the estrogen receptor gene alpha (ESR1) are consistently been associated with bone mineral density (BMD) and fracture.

The purpose of this investigation was to evaluate potential association of single nucleotide polymorphism (SNP) variants of the ESR1 gene and bone mineral density (BMD) of the lumbar spine in Kazakh women.

**Methods:**

140 female participants in Pavlodar clinics with varying measures of BMD. We are examined the potential association of BMD with 2 SNPs from the ESR1 gene (rs2234693 [PvuII] and rs9340799 [XbaI]). Genotyping of the PvuII and XbaI polymorphisms was performed by direct sequencing of the gene fragments containing restriction sites with the identification of genotypes PP, Pp, pp and XX, Xx, xx respectively.

**Results:**

Unadjusted mean BMD values ranged from 1.14±0.14 g/cm^2^ in Caucasian women and 1.03±0.11 g/cm^2^ in Asian women. The association between PvuII polymorphism and BMD at the lumbar spine (p= 0.04 for PP=Pp=pp) was statistically significant in all women. The XbaI polymorphism was not associated with BMD at lumbar spine. The relative risk for low BMD was higher for the marker PvuII (RR=1.51) than for the marker XbaI (RR=1.35).

**Conclusion:**

The PvuII polymorphism had a weak association with lumbar spine BMD. XbaI polymorphism was unlikely to be a predictor of lumbar spine BMD in Kazakh women. These conclusions could help to determine the genetic risk factors for osteoporosis; however, further studies on the association between gene polymorphisms and BMD are needed including larger numbers of participants and genes to clarify genetic risks.

